# Peptides in plant reproduction—small yet powerful

**DOI:** 10.3389/fpls.2025.1506617

**Published:** 2025-02-24

**Authors:** Chun-Lin Yan, Kai-Xun Guan, Hong Lin, Ting Feng, Jiang-Guo Meng

**Affiliations:** Chongqing Key Laboratory of Plant Resource Conservation and Germplasm Innovation, Integrative Science Center of Germplasm Creation in Western China (Chongqing) Science City, School of Life Sciences, Southwest University, Chongqing, China

**Keywords:** plant reproduction, peptide, receptor, pollen tube, fertilization

## Abstract

Peptides, currently being considered as a novel class of plant hormones, play diverse roles in plant growth and development. Peptides trigger signaling by binding to receptors and co-receptors, thus activating cellular signaling pathways. Recently, peptides have been extensively investigated in plant reproduction-related processes, ranging from gametes development to gametes fusion. In this review, we summarize recent advancements related to the role of peptides in plant reproduction and discuss challenges that need to be addressed urgently.

## Introduction

Peptides are short proteins that usually containing no more than 100 amino acid residues (Tavormina et al., 2015). In recent decades, numerous studies have revealed the profound involvement of plant peptides in a wide array of growth and developmental processes, thereby establishing their role as plant hormones (De Coninck and De Smet, 2016; Kim et al., 2021; Olsson et al., 2019). Tomato Systemin was the first peptide hormone identified in plants, based on its role in the immune response (McGurl et al., 1992). Since then, extensive research has led scientists to gain an in-depth understanding of the maturation, classification, evolution, and function of plant peptides (De Coninck and De Smet, 2016; Olsson et al., 2019).

Peptides can be classified into different groups, depending on their synthesis pattern: (1) precursor protein-origin peptides and (2) non-precursor protein-origin peptides. The majority of identified peptides belong to the first group, since they are synthesized from specific prepropeptide precursors. Notably, precursor protein-origin peptides are further classified as Cysteine-Rich Peptides (CRPs) and post-translational modification peptides (Olsson et al., 2019). Many plant species have numerous peptide genes. For example, *Arabidopsis* contains more than 1,000 peptide-coding genes, of which 759 are CRP genes and 431 are Non-CRP genes (Huang et al., 2015). Compared with other genes, CRP genes are more often found in clusters on chromosomes. In most species, 22–39% of the CRP genes are clustered. Considerable collinearities are found between or within species in several syntenic regions containing the CRP genes. Whole-genome duplication is the major force responsible for the expansion of the CRP gene family, with different subfamilies displaying discrepant evolutionary rates, which indicates that these subfamilies are under different selective pressures (Cao and Shi, 2012; Liu et al., 2017).

Sexual plant reproduction necessitates intricate and continuous communication among diverse multicellular organisms (Zhong et al., 2024; Xue et al., 2024). In angiosperms, male and female gametes undergo initiation and development within multicellular structures. Pollen, carrying two sperms, adheres to the stigma, germinates, and transports the sperms to the female gametes through pollen tube polar growth. Subsequently, sperms are released in micropyle, then they fused with the egg cell and central cell separately to develop into embryo and endosperm, respectively. This intricate process involves numerous indispensable peptides-mediated signaling mechanisms (Johnson et al., 2019). In this review, we primarily focus on recent advancements elucidating how peptides regulate this complicated process from gamete maturation to gamete fusion and discuss the current challenges and future prospects of peptide studies related to plant reproduction (view **Table 1** for brief information).

**Table 1:** List of peptides involved in plant reproduction.

Peptide	Organism	Receptor	Function	Reference
TPD1	*Arabidopsis thaliana*	EMS1	Cell differentiation, tapetum development, female gametophyte development	Grelon et al., 2016; Huang et al., 2016a; Huang et al., 2016b; Jia et al., 2008; Yang et al., 2003
TDL1A	*Oryza sativa*	MSP1	Ortholog of *Arabidopsis* TPD1	Nonomura et al., 2003; Zhao et al., 2021; Zhao et al., 2008
MAC1	*Zea mays*	MSP1	Ortholog of *Arabidopsis* TPD1	Wang et al., 2012
CLE19	*Arabidopsis thaliana*	PXL1	Pollen wall formation	Wang et al., 2017b; Yu et al., 2023
CIF3/4	*Arabidopsis thaliana*	GSO	Pollen wall formation	Truskina et al., 2022; Tsuwamoto et al., 2008;
EPFLs	*Arabidopsis thaliana*	ERf	Female gametophyte development	Cai et al., 2023; Franco, 2023; Li et al., 2023b
LAT52	*Solanum lycopersicum*	PRK2	Promoting pollen hydration and germination	Tang et al., 2002
RALF23/33	*Arabidopsis thaliana*	FER/ANJ	Promoting ROS generation and inhibiting pollen hydration	Liu et al., 2021a
PCP-Bs	*Arabidopsis thaliana*	FER/ANJ	Repressing ROS production and initiating pollen hydration	Liu et al., 2021a
RALF1/22/23/33	*Arabidopsis thaliana*	FER/CVY1/ ANJ/HERK1	Preventing undesired pollen tube penetration	Lan et al., 2023a
RALF10/11/12/13/25/26/30	*Arabidopsis thaliana*	FER/CVY1/ ANJ/HERK1	Outcompeting stigmatic RALFs and enabling successful pollen tube penetration	Lan et al., 2023a
SCR/SP11	*Brassica napus*	SRK	Self-incompatibility response	Nasrallah, 2019; Shiba et al., 2001; Takayama and Isogai, 2005; Takayama et al., 2001
RALF4/19	*Arabidopsis thaliana*	ANX1/2, BUPS1/2	Regulating pollen tube growth and integrity	Ge et al., 2019a; Ge et al., 2017; Mecchia et al., 2017
RALF34	*Arabidopsis thaliana*	ANX1/2, BUPS1/2	Competing with RALF4/19, contributing to pollen tube rupture and sperms release	Ge et al., 2017
STIG	*Solanum lycopersicum*	PRK1/2	Regulating pollen tube growth	Huang et al., 2014; Kaothien et al., 2005; Liu et al., 2020
CLE45	*Arabidopsis thaliana*	SKM1/2	Promoting pollen tube growth under high temperature	Endo et al., 2013
PSK	*Arabidopsis thaliana*	PSKR	Pollen tube growth and funicular guidance	Stührwohldt et al., 2015
LUREs	*Torenia fournieri*, *Arabidopsis thaliana*	PRK6, MIK1/2, MDIS1/2	Pollen tube attractant	Okuda et al., 2009; Takeuchi and Higashiyama, 2012; Liu et al., 2013; Takeuchi and Higashiyama, 2016; Wang et al., 2016
XIUQIUs	*Arabidopsis thaliana*	–	Pollen tube attractant	Zhong et al., 2019
TICKETs	*Arabidopsis thaliana*	–	Pollen tube attractant	Meng et al., 2019
NPA1	*Arabidopsis thaliana*	–	Pollen tube attractant	Wang et al., 2024
EA1	*Zea mays*	–	Pollen tube attractant	Marton et al., 2005
RALF6/7/16/36/37	*Arabidopsis thaliana*	FER/ANJ/ HERK1	Pollen tube rupture and Polytubey block	Zhong et al., 2022
ES4	*Zea mays*	–	Pollen tube rupture	Amien et al., 2010
SAL1/2	*Arabidopsis thaliana*	–	Pollen tube attractant, fertilization recovery	Meng et al., 2023
EC1	*Arabidopsis thaliana*	–	Sperm cell activation and gametes fusion	Sprunck et al., 2012

## Gamete development

In flowering plants, gametes initiate from sporophytic cells while being surrounded by multicellular tissues. Importantly, peptides facilitate communication between different cells during the gamete development phase.

**Figure 1:**
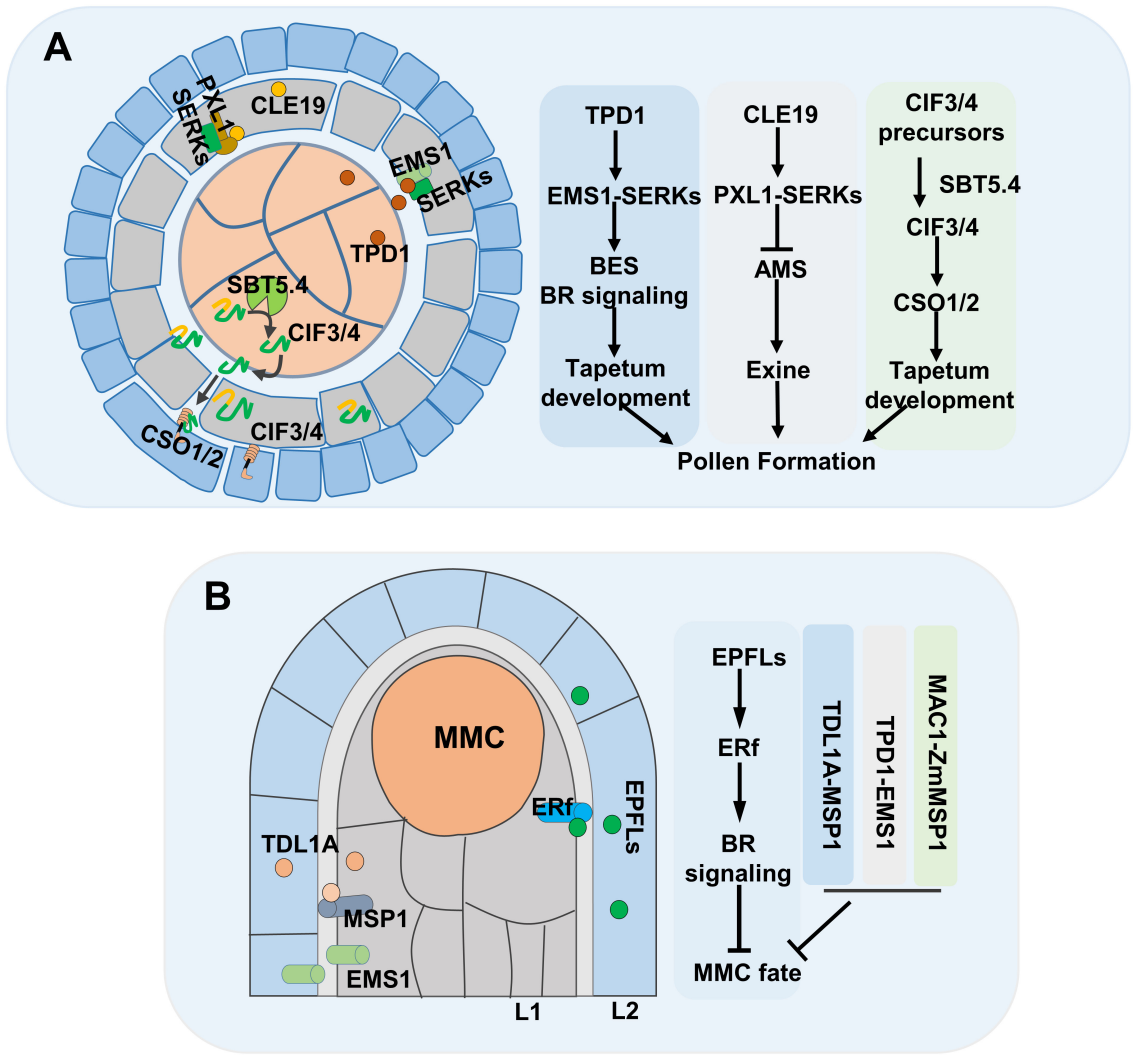
The role of peptides in gametes development. **(A)** Signaling pathway of peptides during the pollen development. **(B)** Signaling pathway of peptides in MMC formation, MMC, megaspore mother cell.

Male gamete development in flowering plants comprises two steps: 1) microsporogenesis, in which somatic cells differentiate into microsporocytes, and 2) male gametogenesis, during which the microsporocyte produces pollen. The microsporocyte is surrounded by a tapetum layer, and frequent material and information exchange between the pollen and the surrounding tapetum layer is essential for successful male gametogenesis (Wilson and Zhang, 2009; Yao et al., 2022). Peptides act as potent signaling molecules during male gametogenesis (**Figure 1A**). In *Arabidopsis*, *TAPETUM DETERMINANT 1* (*TPD1*), encoding a small secreted CRP, is expressed in the microsporocyte and tapetum, and loss-of-function mutations in *TPD1* lead to excess sporocyte production and absence of tapetum within anthers, causing male sterility. Additionally, *EXCESS MICROSPOROCYTES 1* (*EMS1*), encoding a Leucine-Rich-Repeat (LRR) domain receptor kinase, is expressed in tapetum, and the *ems1* mutant is phenotypically similar to the *tpd1* mutant. TPD1 interacts with the extracellular LRR region of EMS1, inducing EMS1 phosphorylation (Grelon et al., 2016; Huang et al., 2016b; Jia et al., 2008; Yang et al., 2003). The *SOMATIC EMBRYO RECEPTOR KINASE1* (*SERK1*) and *SERK2* display the same expression pattern as *EMS1*, and *serk1/2* mutants exhibit a similar phenotype as the *ems1* and *tpd1* mutants. Additionally, SERK1 and SERK2 interact with EMS1 *in vivo* and function as the co-receptors of EMS1, and TPD1 binds to the EMS1-SERK1 heterodimer to phosphorylate EMS1 (Li et al., 2017). Subsequently, it was found that Brassinosteroid (BR) is involved in TPD1-EMS1-SERK1 pathway. BRI1 EMS SUPPRESSOR 1 (BES1) is a key transcription factor specifically regulating BR-mediated gene expression. The null mutants of *BES1* family lack the tapetal layer in anthers, similar to the defect of *tpd1*, *ems1* and *serk1/2*, and gain-of function mutation of *BES1* (*bes1-D*) can significantly suppress the male sterility of *tpd1*, *ems1*, and *serk1/2*. Additionally, EMS1 is necessary for the nuclear localization of BES1. In summary, BES1 mediated BR signaling act downstream of TPD1-EMS1/SERK1 (Chen et al., 2019).

In rice (*Oryza sativa* L.), TPD1-like 1A (TDL1A), an ortholog of TPD1, interacts with MULTIPLE SPOROCYTE1 (MSP1), an ortholog of EMS1. The *ostdl1a* and *msp1* mutants display similar defects in anther phenotype as the *tpd1* and *ems1* mutants. TDL1A acts as a ligand for MSP1 and regulates pollen development (Nonomura et al., 2003; Zhao T. et al., 2021; Zhao X. et al., 2008). In maize (*Zea mays*), MULTIPLE ARCHESPORIAL CELLS 1 (MAC1), which is orthologous to TDL1A, interacts with the *Zm*MSP1 protein to regulate male gametophyte development (Wang et al., 2012). The results of those studies have demonstrated that the function of the TPD1-EMS1 signaling pathway is conserved in different plant species.

Intact pollen wall, consisting of exine (outer wall), intine (inner wall), and pollen coat, plays a crucial role in protecting pollen from various stresses and is essential for pollen germination. The tapetum layer is necessary for pollen wall formation. Tapetum cells express *CLAVATA3/ESR-RELATED 19* (*CLE19*), and loss-of-function and dominant-negative mutants as well as overexpression transgenic lines of *CLE19* exhibit significant male fertility defects. Additionally, *cle19* mutants show abnormal accumulation of pollen exine, indicating that CLE19 signaling is vital for pollen exine formation (Wang et al., 2017b). PXY-LIKE1 (PXL1) is present in both tapetum cells and pollen grains. Anthers of *pxl1-1* and *pxl1-2* mutants display a slight reduction in size with abnormally filled pollen exine. Dominant-negative mutation of *PXL1* can suppress the developmental defects caused by *CLE19* overexpression. Furthermore, physical interaction between CLE19 and PXL1 promotes the phosphorylation of PXL1, confirming its role as a receptor in the CLE19 signal transduction pathway (Yu et al., 2023). SERKs act as co-receptors in multiple Receptor-Like-Kinases (RLKs)-mediated signaling pathways, including the PXL-CLE cascade where they serve as co-receptors alongside PXL to form complexes dependent on CLE19 activity (Yu et al., 2023). These findings contribute to our understanding of a novel mechanism underlying the pollen wall formation process. Tapetum produced sulfo-peptide precursors CASPARIAN STRIP INTEGRITY FACTOR 3 (CIF3) and CIF4 are processed by the pollen localized subtilase *At*SBT5.4, a pollen-specific subtilisin serine protease. Then, these mature CIF3 and CIF4 peptides diffuse between tapetal cells to bind to GASSHO (GSO) receptors located in the middle layer which is a tissue surrounding the tapetum and developing pollen, thereby triggering GSO-dependent tapetum activation and consequently leading to polarized sporopollenin secretion (Truskina et al., 2022; Tsuwamoto et al., 2008).

The initiation of the female germline in most flowering plants occurs in the L2 (subepidermal) layer of ovule primordia, resulting in the formation of a single Megaspore Mother Cell (MMC), which then undergoes a series of developmental processes to generate the female gametophyte (Yang et al., 2010). The L1 (epidermal) layer is capable of secreting signals that restrict MMC formation to a single cell (**Figure 1B**) (Cai et al., 2022). *EPIDERMAL PATTERNING FACTOR-like* (*EPFLs*), encoding CRP ligands, are expressed in the L1 layer, while ERECTA family (ERf) receptor kinases are enriched in the plasma membrane of both L1 and L2 layer cells of ovule primordia. Genetic evidence shows that *epfl1/2/4/6* and *er/erl1/2* mutants exhibit supernumerary enlarged MMC-like cells. Furthermore, EPFLs have been shown to interact with ERf receptor kinases to regulate female germline specification through BR signaling (Cai et al., 2023; Franco, 2023; Li et al., 2023b). The TPD1-EMS1 cascade also plays a role in female gametophyte development. In rice, *TDL1A* and *MSP1* are highly co-expressed in the nucellus epidermis that surrounds the MMC, disruption of TPD1–EMS1-like signaling leads to the formation of extra MMCs in ovules (Zhao et al., 2008). However, in *Arabidopsis* ovule primordia, *TPD1* is weakly expressed at the distal end of ovule integuments; *EMS1* is expressed in the nucellus epidermis; and ectopic expression of *TPD1* causes ovule developmental defects (Huang et al., 2016a).

While the function of peptides in gametes development is under investigation, progress is slow, primarily because of the presence of multiple layers of tissue surrounding the plant gametes. This greatly challenges the study of cellular-level functions of small peptides. Recently, the utilization of single-cell RNA sequencing (scRNA-seq) technology facilitated the construction of comprehensive single-cell transcriptomes during ovule or anther development (Rodriguez-Villalon and Brady, 2019; Song et al., 2020). This advancement will enable us to explore a wider range of peptides involved in gamete developmental processes.

## Pollen–stigma recognition

**Figure 2:**
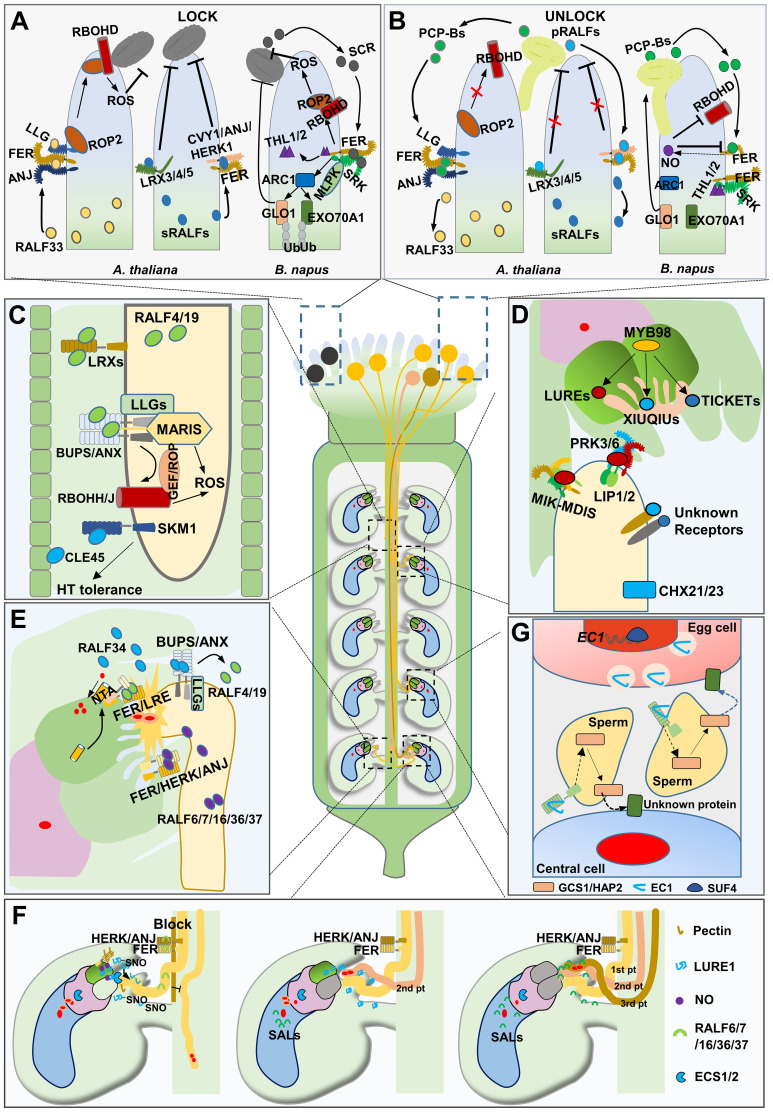
Peptides in pollen/pollen tube-pistil interaction. **(A)** the schematic diagram of “Lock” state on stigma. Incompatible pollen triggers an incompatibility response in the stigma of Brassicaceae. **(B)** the schematic diagram of “Unlock” state on stigma. “Key” peptides from compatible pollen activate the “Lock” system to promote pollen germination. The schematic diagram shows the growth of pollen tube in transmitting tract **(C)** and attractted by the ovule **(D)**. **(E)** the schematic diagram of pollen tube reception. **(F)** Polytubey block and fertilization recovery mechanisms in *Arabidopsis*. **(G)** the schematic diagram of gamete fusion and activation.

In angiosperms, compatible pollen grains that land on the stigma of the pistil undergo a series of processes, including capture, adhesion, hydration, and germination (Dresselhaus and Franklin-Tong, 2013). Frequent information exchange takes place between the pollen and stigma, forming a precise “lock-and-key” mechanism that determines pollen acceptable germination (**Figures 2A, B**) (Cheng and Li, 2023). Pollen Coat Proteins (PCPs), produced in the pollen coat, play crucial roles in this “lock-and-key” system (Wang et al., 2017a). In tomato (*Solanum lycopersicum*) plants, the pollen autocrine peptide LATE ANTHER TOMATO52 (LAT52) interacts with the pollen-specific protein POLLEN-SPECIFIC RECEPTOR KINASE2 (*Le*PRK2), promoting pollen hydration and germination (Tang et al., 2002). In the self-compatible (SC) species *Arabidopsis thaliana*, papilla cell produced peptides RAPID ALKALINAZATION FACTOR23/33 (RALF23/33) bind to the FERONIA/ANJEA/LORELEI-like-GPI-anchored protein (FER/ANJ–LLG1) complex, activating the RopGEF–ROP–RBOHD signaling pathway and promoting ROS generation, forming a “lock” to inhibit pollen hydration. Upon compatible pollen landing, pollen-secreted “key” peptides, including PCP-Bs (*At*PCP-Bα, *At*PCP-Bβ, *At*PCP-Bγ, *At*PCP-Bδ), compete with RALF23/33 to bind to FER/ANJ, thereby repressing ROS production and initiating pollen hydration (Liu et al., 2021a). Recently, a similar mechanism was reported in which FERONIA/CURVY1/ANJEA/HERCULES RECEPTOR KINASE 1 (FER/CVY1/ANJ/HERK1) and cell wall proteins LEUCINE-RICH REPEAT EXTENSIN3/4/5 (LRX3/4/5) on papilla cell surfaces interact with autocrine stigmatic RALF1/22/23/33 peptide ligands (sRALFs), establishing a “lock” that prevents undesired pollen tube penetration. Compatible pollen-derived RALF10/11/12/13/25/26/30 peptides (pRALFs) act as a “key” by outcompeting sRALFs and enabling successful pollen tube penetration. Furthermore, researchers have utilized pRALFs as mentors to breakdown the lock system and facilitate interspecific pollen tube penetration and fertilization (Lan et al., 2023a). In the self–incompatible (SI) plant species *Brassica napus*, S–LOCUS CYSTEINE RICH PROTEIN/S-LOCUS PROTEIN11 (SCR/SP11), secreted from the incompatible pollen and recognized by the papilla cell-localized protein S-LOCUS RECEPTOR KINASE (SRK), determines the self-incompatibility response (Nasrallah, 2019; Shiba et al., 2001; Takayama and Isogai, 2005; Takayama et al., 2001). M-LOCUS PROTEIN KINASE (MLPKs), acting as co-receptors of SRK, play positive roles in self-incompatibility mechanisms (Kakita et al., 2007). Additionally, THIOREDOXIN H-LIKE proteins (THL1 and THL2) interact with SRK to inhibit its kinase activity, and SCR/SP11 competitively interact with SRK to release THL1/2, resulting in the activation of the downstream gene *ARMADILLO-REPEAT-CONTAINING1*(*ARC1*), encoding an E3 ubiquitin ligase in stigma cells. This leads to the rejection of SI pollen through the degradation of germination-promoting proteins such as GLYOXALASE 1 (GLO1) and EXO70A1 (Goring et al., 2014; Indriolo et al., 2012; Yamamoto and Nasrallah, 2013). More recently, the classical SCR-SRK cascade has been further expanded. SCR-SRK can recruit the receptor protein FER, which activates the production of ROS in stigma cells, and high ROS levels inhibit pollen germination, thereby aiding in the rejection of self-pollen or interspecific pollen (Huang et al., 2023; Zhang et al., 2021). When intraspecific pollen landing on the stigma of *Brassica napus*, PCP-Bs from pollen coat could trigger Nitric Oxide (NO), which nitrosates FER and RBOHD/F to suppress ROS production in stigmas to facilitate intraspecific pollen germination and growth (Huang et al., 2023; Zhang et al., 2021).

Interspecies hybridization is a powerful tool for hybrid breeding; however, one of the major challenges lies in the reproductive barriers present on stigma. Leveraging the elucidated knowledge of the “lock-and-key” system, researchers have developed specific mutants, such as *fer-4*, *srk*, and *anj*, and chemical compounds, including ROS scavenger Na-SA, NO donor and SRK disruptor AS-ODN, to interfere with this system at the stigma level (Huang et al., 2023). The mentoring effect of pRALFs has been employed by researchers to overcome compatibility barriers by allowing both incompatible and compatible pollen to deceive the “lock” mechanism, thereby facilitating the germination and penetration of otherwise incompatible pollen grains (Lan et al., 2023a). Additionally, certain abiotic factors play significant roles in determining pollen tube penetration; for instance, high temperatures disrupt the targeting of SRK to plasma membranes, leading to weakened self-incompatibility response in plants (Yamamoto et al., 2019). Similarly, application of the NaCl solution and other saline water to field-cultivated *Brassica napus* plants facilitates self-pollination in this SI species, although the underlying molecular mechanisms remain unknown (Li et al., 2024).

## Pollen tube growth and guidance

After pollen germination and penetration, the pollen tube grows through the style into the transmitting tract and then emerges from the transmitting tract, targeting the ovule micropyle (Li et al., 2018). Peptide signals play a key regulatory role in keeping growth and guidance of the pollen tube in different plant species (**Figures 2C, D**). In solid and enclosed pistil tissues, integrity of the pollen tube is a prerequisite for its polar growth. In *Arabidopsis*, RALFs, together with *Cr*RLK1L homologs, and LLGs form signaling modules that are essential for preserving pollen tube integrity until it is accepted by female gametes (Zhou et al., 2024). RALF4/19 are pollen-expressed proteins, *ralf4/19* pollen tubes germinated normally *in vitro*, but their pollen tubes burst prematurely *in vivo*, producing abnormally short siliques and were male sterile. The *CrRLK1L* homologs *ANXUR1/2* (*ANX1/2*) and *BUDDHA’S PAPER SEAL 1/2* (*BUPS1/2*) are highly expressed paralogs of *FER* specifically in pollen grains. In *anx1/2* and *bups1/2* mutants, pollen tubes ruptured prematurely during growth within pistils, leading to pollen tube growth arrest, the loss of either pair of *CrRLK1L* homologs leads to male sterility, similar to *ralf4/19*. Biochemical assays revealed that ANXs and BUPSs form a heteromer, all four receptor kinases (i.e., ANX1/2 and BUPS1/2) interact with LLG2/3 to form a large complex, while RALF4/19 act as ligands of the ANXs–BUPSs–LLGs complex (Feng et al., 2019; Ge et al., 2017, 2019a, 2019b, 2019c). COBRA-like protein 11 (COBL11) interacts with RALF4/19, ANX1/2, and BUPS1/2, and the functional loss of COBL11 disrupts the proper distribution of RALF4/19 and the membrane localization of ANXs (Li et al., 2023a). MARIS and RBOH oxidases (RBOHH/J) act downstream of the tripartite complexes, indicating the involvement of ROS in maintaining pollen tube integrity (Boisson-Dernier et al., 2015; Kaya et al., 2014; Liao et al., 2016). The application of RALF4/19 on pollen could inhibits pollen germination and growth, pollen-expressed LRX8/9/10/11 interact directly with RALF4/19 to control their growth-inhibitory function, suggesting that the RALF-LRX module works as a brake to regulate pollen tube growth (Mecchia et al., 2017; Moussu et al., 2020; Sede et al., 2018). Interestingly, RALF4/19 orchestrate both pollen tube growth and integrity, and slower growth appears to be better for integrity. However, the connection between ANX–BUPS–RALFs and LRXs–RALFs mechanisms remains unclear. In maize, *Zm*RALFs mediate both pollen tube integrity and growth through the FER–like receptor kinases, *Zm*LLGs and extension–like proteins (Zhou et al., 2024). Additionally, RALFs in regulating pollen tube integrity is also observed in rice (Kim et al., 2023). Together, these studies suggest that the RALFs–controlled mechanism of pollen tube integrity maintenance during growth is considerably conserved in different plant species. Besides RALFs, other peptides are also involved in regulating pollen tube growth. In tomato plants, *Le*STIG, a small cysteine-rich protein from pistil, replaces LAT52 to bind to *Le*PRK1/2 receptors, thus activating RAC/ROP GTPases for pollen tube growth regulation (Huang et al., 2014; Kaothien et al., 2005; Liu et al., 2020). Under heat stress, pistils secrete peptides, such as CLE45, which bind to STERILITY-REGULATING KINASE MEMBER1 (SKM1) and SKM2 receptors, promoting the growth of pollen tube through the pistil while protecting sperm delivery from high temperatures (Endo et al., 2013).

The final destination of pollen tubes is the ovule micropyle; however, before reaching the micropyle, pollen tubes must leave the transmitting tract, travel through the septum and grow along the funiculus. This process can be divided into two specific steps (Wang et al., 2023). In the first step, the pollen tube needs to emerge from the transmitting tract and elongate to the funiculus, also known as funicular guidance. The pollen tube puncture points are uniformly distributed across the septum, suggesting that pollen tube emergence is not random and that specific mechanisms must regulate the funicular guidance. Recent imaging analysis shows that this emergence from transmitting tract depends on sporophytic signals from the ovule (Mizuta et al., 2024).

K^+^ transporters CATION/PROTON EXCHANGERS 21/23 (CHX21/23) are proteins specific to pollen tubes. Loss of these proteins in *chx21/23* results in pollen tubes being unable to emerge out of the transmitting tract. *In vivo* experiments show that *chx21/23* pollen tubes fail to target the ovule correctly (Lu et al., 2011). These results indicate that CHX21/23 potentially respond to unknown signals involved in pollen tube emergence from transmitting tract. In *Arabidopsis*, Mitogen-Activated Protein Kinase (MPK3) and MPK6, which are involved in various biotic and abiotic stress responses, are enriched in pollen tube nuclei. Pollen tubes of the *mpk3/6* mutant were observed to be defective in targeting funiculus (Guan et al., 2014). Phytosulfokine (PSK), a disulfated pentapeptide that is processed from a 90–100 amino acid precursor, further catalyzed by a tyrosylprotein sulfotransferase (TPST) in trans-Golgi, and PSK is perceived by LRR receptor PSKR (Matsubayashi and Sakagami, 1996; Yang et al., 1999; Komori et al., 2009; Matsubayashi et al., 2002). In *Arabidopsis*, PSKs, TPST, and PSKRs are expressed in both pollen tubes and pistil tissues. Phenotypic analyses of *tpst-1* and *pskr1-3 pskr2-1* mutants revealed that the PSK signaling pathway in pollen tubes and maternal tissues plays a crucial role in funicular guidance (Stührwohldt et al., 2015). While CHXs, MPKs, and PSK signaling pathways have been implicated in funicular guidance, several questions remain unresolved. Specifically, it is unclear whether CHXs and MPKs are involved in the PSK pathway and whether PSK serves as a direct signal for controlling funicular guidance. The current priority is to identify a direct signal that regulates funicular guidance. Previous research has indicated that pollen tube attraction signals produced by embryo sac are short-range and may not reach the surface of the guiding tissue (Mizuta and Higashiyama, 2018; Li et al., 2018). In contrast, funiculus or ovule integument, which are closer to the point where pollen tube penetrates the septum, may generate signals that directly influence pollen tube’s exit from transmitting tract and its subsequent guidance towards funiculus.

The second step is ovular guidance, with the pollen tube elongating from funicular to micropyle opening. A series of peptides secreted from synergid cells guide the pollen tube to the micropyle. *Tf*LUREs is the first group of peptides identified as pollen tube attractants in *Torenia fournieri*. *Tf*LUREs are defensin-like CRPs secreted by ovule synergid cells that diffuse towards the micropylar and funicular region to attract pollen tubes (Okuda et al., 2009). *Arabidopsis* contains seven homologs of *Tf*LUREs (Okuda and Higashiyama, 2010; Takeuchi and Higashiyama, 2012). Pollen tube tip-localized Receptor-like Kinase 6 (PRK6) interacts with LURE1 through its extracellular LRR domain, *in vitro*, *prk6* pollen tubes fail to target LURE peptides. Additionally, two receptor-like cytoplasmic kinases (RLCKs), LOST IN POLLEN TUBE GUIDANCE1 (LIP1) and LIP2, are also required for LURE-mediated pollen tube guidance. Furthermore, LIP1/2 could directly interact with PRK6, suggesting that PRK6-LIP1/2 act as a receptor complex for LUREs (Liu et al., 2013; Takeuchi and Higashiyama, 2016). Recent reports have indicated that PRKs can interact with ROPGEFs, which activate RAC/ROPs, playing a crucial role in polar cell growth, further expanding PRK6 signaling (Gu et al., 2006; Takeuchi and Higashiyama, 2016; Luo et al., 2017).

LRR-RLKs MDIS1-INTERACTING RECEPTOR LIKE KINASE 1/2 (MIK1/2) and MALE DISCOVERER 1/2 (MDIS1/2) are membrane proteins located at the tip of pollen tube. Pollen tubes lacking *mik1/2* and *mdis1* show reduced sensitivity to LURE1, indicating that the MIKs-MDISs complex serves as another receptor for LURE1 (Wang et al., 2016). The PRK6-LURE complex, which mediates pollen tube guidance, also functions as a prezygotic isolation barrier. Wild-type *Arabidopsis* ovules preferentially attract conspecific pollen tubes, but septuple *atlure* mutant ovules can attract a significant amount of *Arabidopsis lyrata* pollen tubes. This suggests that *At*LURE1/PRK6-mediated signaling promotes conspecific micropylar pollen tube attraction (Liu et al., 2021b). Interestingly, the *atlure* mutant displays normal fertility, suggesting the existence of other attractants. Currently, the LURE-mediated pollen tube guidance mechanism appears to be relatively well-established; however, several unresolved issues remain. First, structural studies have shown that a C-terminal loop of the LRR domain is responsible for the recognition of *At*LURE1.2. This recognition is mediated by a set of residues that are largely conserved among PRK6 homologs from *Arabidopsis lyrata* and *Capsella rubella* but not in *Torenia fournieri*, where the first LURE was identified, which introduces some confusion (Zhang et al., 2017).

The defensin-like peptides XIUQIUs and TICKETs have been found to possess pollen tube attractant activity (Meng et al., 2019; Zhong et al., 2019). Specifically, the nonspecies-specific attractant activity of XIUQIUs is independent of PRK6, although the XIUQIU receptor remains unclear (Zhong et al., 2019). Recently, species-specific pollen tube attraction activity was identified among sister species of the Brassicaceae family through the discovery of NPA1 (Wang et al., 2024), a synergid-secreted non-defensin-like peptide that contributes to species barriers. Interestingly, MYB98 transcription factor regulates the expression of all aforementioned attractants in synergid cells (Kasahara et al., 2005; Meng et al., 2019; Punwani et al., 2007; Takeuchi and Higashiyama, 2012; Zhong et al., 2019); however, offspring production still occurs in *myb98* mutants, indicating the presence of non-synergid cell-mediated or non-MYB98-regulated attractant signaling. In maize, an egg apparatus is developed to mediate pollen tube guidance. The egg-apparatus-secreted polymorphic peptide *Zm*EA1 exhibits pollen tube attractant activity, indicating that the egg cell, or potentially other types of cells, is also capable of secreting attractants (Marton et al., 2005). Intriguingly, when *ZmEA1* is expressed in *Arabidopsis* synergid cells, the secreted *Zm*EA1 enables *Arabidopsis* ovules to attract maize pollen tubes *in vitro* towards the micropylar opening (Marton et al., 2012).

## Pollen tube reception

After entering the micropyle, the pollen tube ruptures by interacting with synergid cells (**Figure 2E**). During this interaction, one of the two synergid cells undergoes elimination and the synergid cell that persists is called persistent synergid cell (Maruyama et al., 2015).

RALF4/19 is responsible for maintaining the integrity of pollen tubes. However, when the pollen tube arrives at the micropyle, ovule-derived RALF34 competes with RALF4/19 to bind to BUPS1 and ANX1. The replacement of RALF4/19 by RALF34 disrupts pollen tube integrity, leading to pollen tube rupture and sperm release (Ge et al., 2017). Five pollen-expressed RALFs (RALF6/7/16/36/37) are involved in the bursting of pollen tubes, as evident from the failure of pollen tubes to burst in the *ralf6/7/16/36/37* quintuple mutant. *FER*, *HERK*, and *ANJ* are expressed in ovules and redundantly participate in pollen tube reception; the pollen tubes of *fer* and *anj herk1* mutants fail to burst and show overgrowth in the micropylar region. Furthermore, FER, HERK, and ANJ physically interact with each other, and RALF6/7/16/36/37 proteins function as ligands for the FER-HERK-ANJ complex (Zhong et al., 2022). RALF4/19 also act as ligands for the FER-LRE complex and enhance the Ca^2+^ channel activity of NORTIA (NTA) in synergid cells, which is essential for pollen tube reception; however, the mechanisms underlying Ca^2+^-induced rupture of pollen tube and elimination of one synergid cell remain unclear (Gao et al., 2023, 2022). In maize, pollen tube plasma membrane-localized potassium channel KZM1 is activated by the synergid cell-secreted peptide EMBRYO SAC4 (*Zm*ES4). The interaction between *Zm*ES4 and KZM1 induces K^+^ influx, resulting in altered osmotic pressure that promotes water uptake and potentially leads to pollen tube rupture (Amien et al., 2010).

## Polyspermy block and fertilization recovery

Polyspermy is a phenomenon where multiple sperm cells enter a single egg cell. This leads to aberrant chromosome numbers and consequently failed embryo development. In angiosperms, polyspermy always occurs when multiple pollen tubes enter the same ovule. Plants have evolved various strategies to prevent polyspermy (**Figure 2F**).

Once the pollen tube penetrates the transmitting tract, a local block is established. In *Arabidopsis*, pollen tube-derived peptides RALF6/7/16/36/37 interact with septum-localized receptors FER/ANJ/HERK to establish the early polyspermy block, inhibiting the local emergence of other pollen tubes from the transmitting tract, however, what happens downstream the RALFs-FER/ANJ/HERK remains unknown (Zhong et al., 2022). After the pollen tube ruptures in the female gametocyte, RALFs cease to exist and the block is temporarily lost, creating an opportunity for another pollen tube to emerge and target the same ovule. To avert polyspermy at this stage, the pollen tube, after reaching the micropyle, induces the production and accumulation of NO at the filiform apparatus in a process that is dependent on FER and mediated by de-esterified pectin. NO inhibits the activity of the already secreted LUREs and prevents further secretion of these attractants from synergid cells by nitrosating both precursor and mature forms of LUREs (Duan et al., 2020). After fusing with the sperm, the egg cell secretes two aspartic proteases, ECS1/2, that specifically cleave the pollen tube attractants LUREs (Yu et al., 2021). However, if the fertilization conducted by the first pollen tube fails, ovules must attract another pollen tube to achieve successful fertilization, this process is known as fertilization recovery. Plants need to coordinate fertilization recovery with the polyspermy block. RALFs-induced polyspermy block expired after the rupture or fertilization defect of the first pollen tube, allowing another pollen tube to emerge and target the ovule (Zhong et al., 2022). The persistent synergid cell continues to secrete attractants towards the micropylar and funicular region in order to guide the pollen tube (Kasahara et al., 2012, 2013; Maruyama and Higashiyama, 2016; Maruyama et al., 2015). However, it is still unknown how the NO-mediated polyspermy block expired. Recently, it was discovered that central cells play a crucial role in fertilization recovery. When both synergid cells are eliminated or defected, the central cell secretes CRPs SALVAGER1 (SAL1) and SAL2, which function to attract pollen tubes. This backup mechanism may explain why *myb98* and *atlure xiuqiu* mutants are fertile (Dresselhaus and van der Linde, 2023; Lan et al., 2023b; Meng et al., 2023, 2024).

## Gamete fusion and activation

After two sperm cells are released, they migrate separately towards the central cell and egg cell. Subsequently, one sperm fuses with egg cell to initiate embryo formation, while the second sperm fuses with central cell to facilitate endosperm development. Despite limited knowledge regarding gamete fusion and activation, EGG CELL1 (EC1) peptides have been identified as crucial players in this process. In *Arabidopsis*, five *EC1-like* genes (*EC1.1*–*EC1.5*) are specifically expressed in egg cell, and EC1 peptides accumulate in storage vesicles prior to sperm arrival. Upon contact with sperm cells, exocytosis occurs within the EC1 vesicles. When EC1 peptides were applied to sperm cells, the protein HAPLESS2/GENERATIVE CELL SPECIFIC1 (HAP2/GCS1), essential for gamete fusion, redistributes from the endomembrane system to the cell surface in order to facilitate gamete fusion. Furthermore, ovules lacking EC1 exhibit a blockage in gamete fusion (Sprunck et al., 2012). These findings suggest that EC1 promotes the ability of sperm to fuse with egg cell (**Figure 2G**); however, the receptor of EC1 remains a significant unresolved mystery. The C2H2 transcription factor SUPPRESSOR OF FRIGIDA4 (SUF4) directly regulates the expression of *EC1* genes, and *suf4-1* mutant exhibits a moderate *ec1* phenotype, indicating that SUF4 controls sperm fusion by regulating *EC1* expression (Resentini et al., 2017).

## The potential application of peptides research

**Figure 3:**
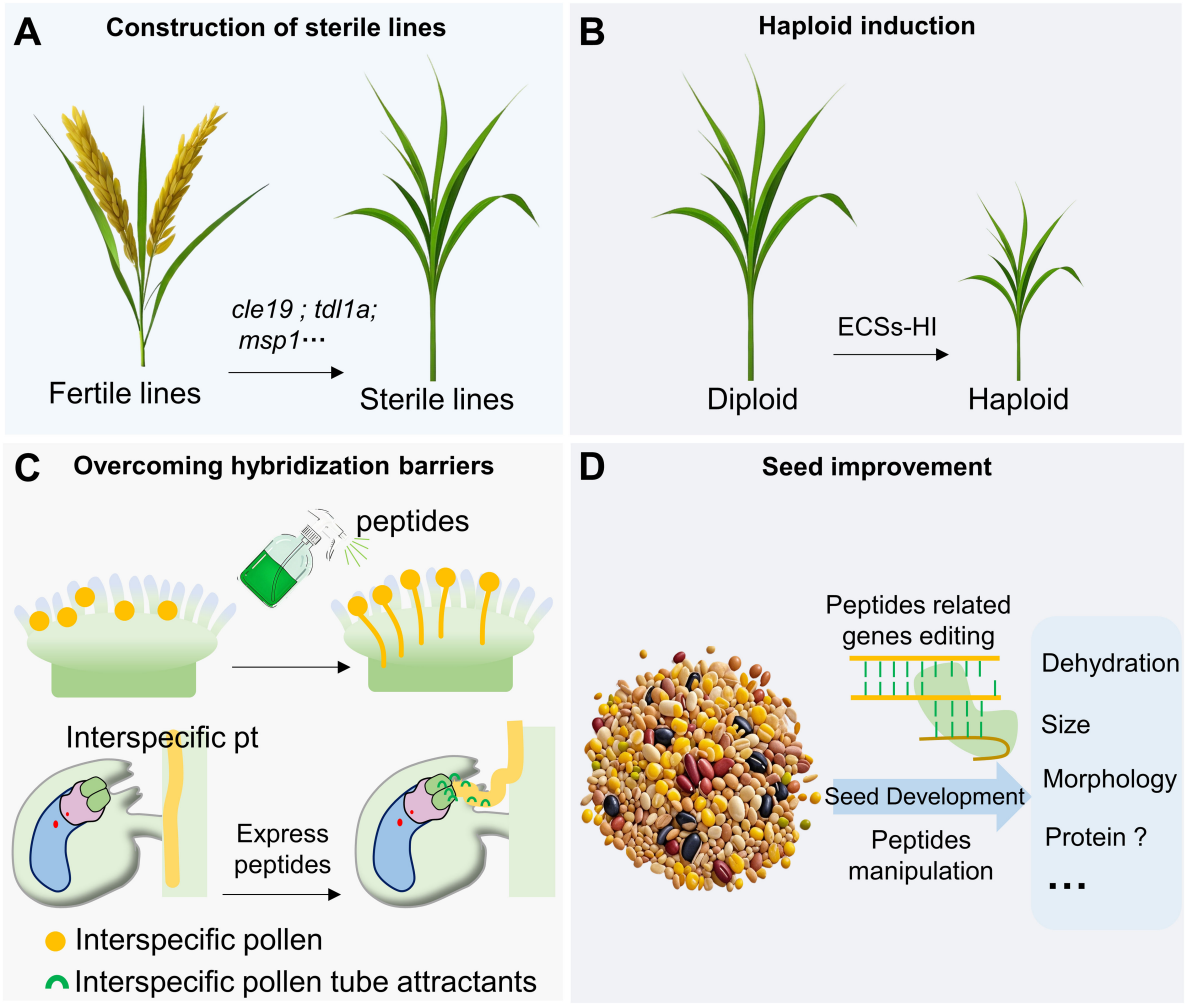
Potential application of peptides research. Peptide studies in plant reproduction are potentially applicable to the construction of sterile lines **(A)**, haploid induction **(B)**, overcoming hybridization barriers **(C)** and seed improvement **(D)**.

Despite the increasing attention given to small peptides, the practical application of this research remains a challenging issue. However, there is considerable potential for certain aspects of this knowledge to be applied in agricultural breeding in the future (**Figure 3**). Many peptides play critical roles in gametic development, aiding in the construction of sterile lines in various crops to accelerate breeding processes (Erbasol Serbes et al., 2019; Liu et al., 2023; Xiong et al., 2023). The *ecs1/2* double mutant can induce maternal haploids in *Arabidopsis*, and *ECS* mutations are also capable of producing haploids in rice. Given that ECS homologs exist in other species such as *Brassica napus* and *Raphanus sativus*, it suggests that ECSs-HI (ECSs-haploid induction) technology can be widely utilized in agricultural breeding (Mao et al., 2023; Zhang et al., 2023).The identification of “key” peptides on the stigma and the investigation of pollen tube attractants can help overcome reproductive barriers and facilitate the creation of distant hybrid species (Marton et al., 2012; Uebler et al., 2013; Wang et al., 2016; Zhong et al., 2019, 2022; Liu et al., 2021a; Liu et al., 2021b; Huang et al., 2023). Several investigations have indicated that specific peptides participate in seed development and consequently adjust the size and morphology of seeds (Jin et al., 2016; Sui et al., 2016). Recent studies have revealed that the peptide microRPG1 regulates the dehydration process of corn grains during seed maturation (Yu et al., 2024). Manipulating microRPG1 to alter the dehydration rate of corn grains holds significant potential for developing varieties that are more suitable for easy harvesting.

## Perspectives

Since the initial investigation of plant sexual reproduction, a plethora of findings have been uncovered, with the discovery of peptide functions standing out as one of the most significant breakthroughs (Kim et al., 2021; Qu et al., 2015). However, several aspects of peptides and their role in plant reproduction remain unaddressed and require dedicated pursuit by researchers. First, it will be important to identify new functional peptides involved in plant reproduction. In *Arabidopsis*, 139 and 390 CRPs have been identified in male and female gametophytes, respectively (Huang et al., 2015); however, only a limited number of CRPs have been proven to play crucial roles, leaving significant gaps. Furthermore, no non-precursor protein-derived peptides associated with plant reproduction have yet been identified, posing a major challenge for researchers. Second, the identification of receptors for these peptide ligands remains challenging. The receptors for XIUQIUs, TICKETs, and SALs are currently unknown, more than 600 receptor-like kinases were identified in *Arabidopsis*, and it is difficult to find the specific ligand-receptor combinations (Baudino et al., 2001; Shiu and Bleecker, 2003). Artificial intelligence-based protein interaction prediction tools may facilitate receptor identification. Third, the regulatory mechanisms governing peptide movement remain unclear, including the redistribution of LUREs from the cytoplasm to the filiform apparatus during ovule maturation and the secretion of SALs from the central cell to the micropyle after the elimination of both synergid cells (Meng et al., 2023; Susaki et al., 2022). Finally, the RALFs-FER-ANJ-HERK1 cascade constructs the polytubey block system after pollen tube penetrates through the septum (Zhong et al., 2022). However, what are the precise factors downstream of this system that most directly prevent other pollen tubes from perforating? Are they still peptides? For a long time, it has been postulated that there exists a pollen tube repelling signal, and NO is regarded as a pollen tube repellent (Prado et al., 2004; 2008; Wong et al., 2020). Emerging results have revealed that the production of NO is dependent on FER, is NO a downstream effector of the polytubey mechanism? Additionally, pollen tube itself also generates NO, does the pollen tube directly repel other pollen tubes by releasing NO and participate in the establishment of polytubey block (Duan et al., 2020; Huang et al., 2023; Šírová et al., 2011)? Although these questions present tremendous challenges for us, current discoveries and continuously updated technologies encourage us to carry out further exploration in order to create a comprehensive framework of the regulatory network for plant reproduction.
